# Microscopic reproductive characters enable discrimination of cattail taxa in a highly admixed hybrid zone

**DOI:** 10.1093/aobpla/plag035

**Published:** 2026-08-14

**Authors:** Joanna Freeland, Olivia Kowalczyk, Vikram Bhargav, Heather Wilcox, Gregory Melvin, Jeff Bowman, Carrie Sadowski, Mark Browning, Avery Chambers, Braidy Chambers, Marcel Dorken

**Affiliations:** Department of Biology, Trent University, 1600 West Bank Drive, Peterborough, Ontario, Canada K9L 0G2; Environmental and Life Sciences Graduate Program, Trent University, 1600 West Bank Drive, Peterborough, Ontario, Canada K9L 0G2; Environmental and Life Sciences Graduate Program, Trent University, 1600 West Bank Drive, Peterborough, Ontario, Canada K9L 0G2; Environmental and Life Sciences Graduate Program, Trent University, 1600 West Bank Drive, Peterborough, Ontario, Canada K9L 0G2; Department of Biology, Trent University, 1600 West Bank Drive, Peterborough, Ontario, Canada K9L 0G2; Environmental and Life Sciences Graduate Program, Trent University, 1600 West Bank Drive, Peterborough, Ontario, Canada K9L 0G2; Environmental and Life Sciences Graduate Program, Trent University, 1600 West Bank Drive, Peterborough, Ontario, Canada K9L 0G2; Ontario Ministry of Natural Resources, 2140 East Bank Drive, Peterborough, Ontario, Canada K9J 7B8; Ontario Ministry of Natural Resources, 2140 East Bank Drive, Peterborough, Ontario, Canada K9J 7B8; Ontario Ministry of Natural Resources, 2140 East Bank Drive, Peterborough, Ontario, Canada K9J 7B8; Department of Biology, Trent University, 1600 West Bank Drive, Peterborough, Ontario, Canada K9L 0G2; Department of Biology, Trent University, 1600 West Bank Drive, Peterborough, Ontario, Canada K9L 0G2; Department of Biology, Trent University, 1600 West Bank Drive, Peterborough, Ontario, Canada K9L 0G2; Environmental and Life Sciences Graduate Program, Trent University, 1600 West Bank Drive, Peterborough, Ontario, Canada K9L 0G2

**Keywords:** *Typha latifolia*, *Typha angustifolia*, *Typha* x *glauca*, hybrid zone, advanced-generation hybrids, back-crossed hybrids, bracteoles, pollen, inflorescence, genotypes

## Abstract

Understanding and managing invasive taxa requires accurate identification, which can be challenging when the invader is an interspecific hybrid. An example of this is the hybrid cattail *Typha × glauca*, which is found in a widespread and expanding hybrid zone in North America along with its parent species *Typha latifolia* and *Typha angustifolia*. In some regions, this hybrid is considered invasive because it dominates wetlands, reduces biodiversity, and alters ecosystem functioning. It is also a complex hybrid zone that includes advanced-generation hybrids and hybrid backcrosses to both parent species (hereafter collectively referred to as non-F1 hybrids). Previous studies have shown that gross morphological characters of non-F1 hybrids overlap with those of their parent species, and this can hinder identification and hence monitoring and management programmes in areas of high non-F1 abundance. Here, we show that microscopic pollen and bracteole characteristics are overall highly reliable for taxonomic identification, as they agreed with the genotype-based taxonomic assignments for 409/426 (96%) and 232/244 (95%) of plants, respectively, with non-F1 hybrids the most common source of error. Although these characteristics are limited to flowering plants, and in the case of pollen to a relatively short period of time, they are relatively easy to apply; however, the abundance of *T. latifolia* may be underestimated if plants are identified from inflorescence traits. Nevertheless, this low-cost method for distinguishing cattail taxa could facilitate research into this expanding hybrid zone, plus targeted management of hybrid cattails in areas where they dominate wetlands and disrupt ecosystems.

## Teaser

Understanding biological invasions requires accurate taxonomic identification, and this can be difficult for invasive hybrids that resemble their parents. Cattails (*Typha* spp.) are widespread wetland plants, and in North America, they include invasive hybrids (*Typha × glauca*) that can reduce biodiversity and alter ecosystem processes. We show that microscopic examination of pollen and small flower structures (bracteoles) correctly identified cattail types ∼95% of the time. This inexpensive method can help scientists and land managers monitor and control invasive hybrid cattails.

## Introduction

Macrophytes are essential to the structure and functioning of aquatic ecosystems: they stabilize sediments, alter water levels and stream hydraulics, influence nutrient cycling, and provide habitat for fauna (Ibelings et al. 2007, Christie et al. 2009, O’Brien et al. 2014, Choudhury et al. 2018, Thomaz 2023). However, non-native macrophytes can be detrimental to wetlands if they reduce native biodiversity or alter ecosystems, e.g. by modifying nutrient cycling or water levels (Schultz and Dibble 2012, Zedler and Kercher 2004). Managing invasive macrophytes is seldom straightforward and in some cases is confounded by cryptic invasions, which arise when invasive taxa are superficially similar to native taxa.

**Figure 1:**
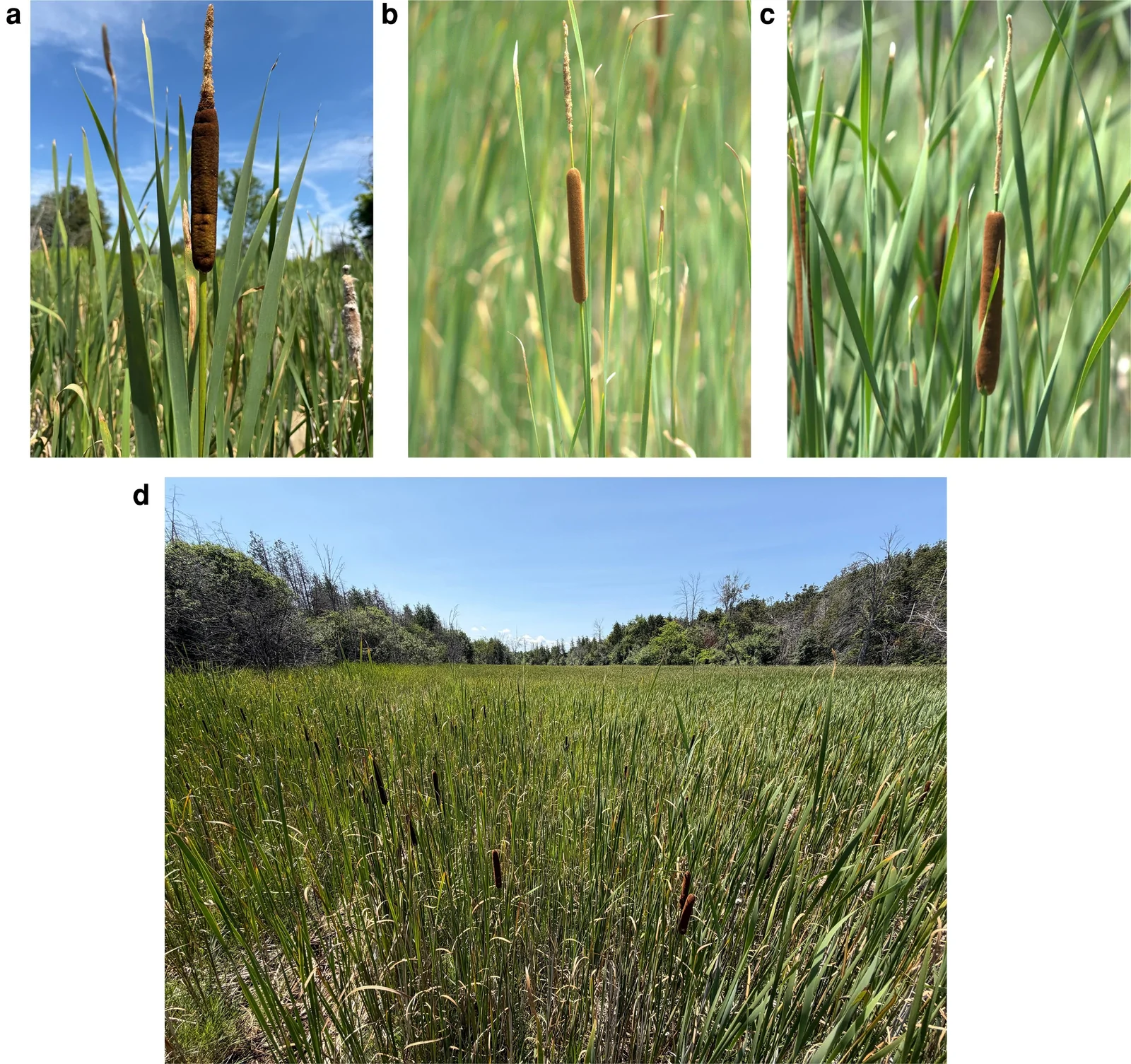
a) *Typha latifolia* is characterized by relatively wide leaves and no gap between male and female inflorescences. b) *Typha angustifolia* is characterized by relatively narrow leaves and a relatively large gap between male and female inflorescences. c) This may be an F1 hybrid, characterized by intermediate leaf widths and inflorescence gap; however, advanced-generation and back-crossed hybrids collectively show a wide range of leaf widths and inflorescence gaps and can be difficult to identify in the field. d) A typical North America *Typha* wetland habitat. *Typha* also grows in ditches, along riverbanks, around lakes and ponds, etc.

*Typha* is a common and globally widespread macrophyte genus foundational to wetlands, with two of the most widely distributed species occurring in North America: *Typha latifolia* and *Typha angustifolia* (Grace and Harrison 1986, Smith 2000). These can hybridize to form *T. × glauca* (Grace and Harrison 1986, Smith 2000), which is considered invasive across large parts of its North American range where it dominates wetlands, alters ecosystem functioning, and reduces native biodiversity (Tuchman et al. 2009, Larkin et al. 2012, Lishawa et al. 2014, Lawrence et al. 2016, 2017) (Fig. 1). The history of this hybrid zone is unclear because of morphological similarities between parent species and hybrids. However, a study published in 1952 that used detailed morphological characters to identify 821 *Typha* specimens collected across the northeastern USA concluded that there were more hybrids (∼24%) than *T. angustifolia* (∼10%) (Fassett and Calhoun 1952). In 1999, Kuehn and White (1999) concluded that the prevalence of hybrids was likely underestimated in regions around the Laurentian Great Lakes (LGL), based on higher morphological variability in hybrids compared to parent species. The range expansion of *T. × glauca* therefore predated our awareness of this now widespread taxon.

**Figure 2:**
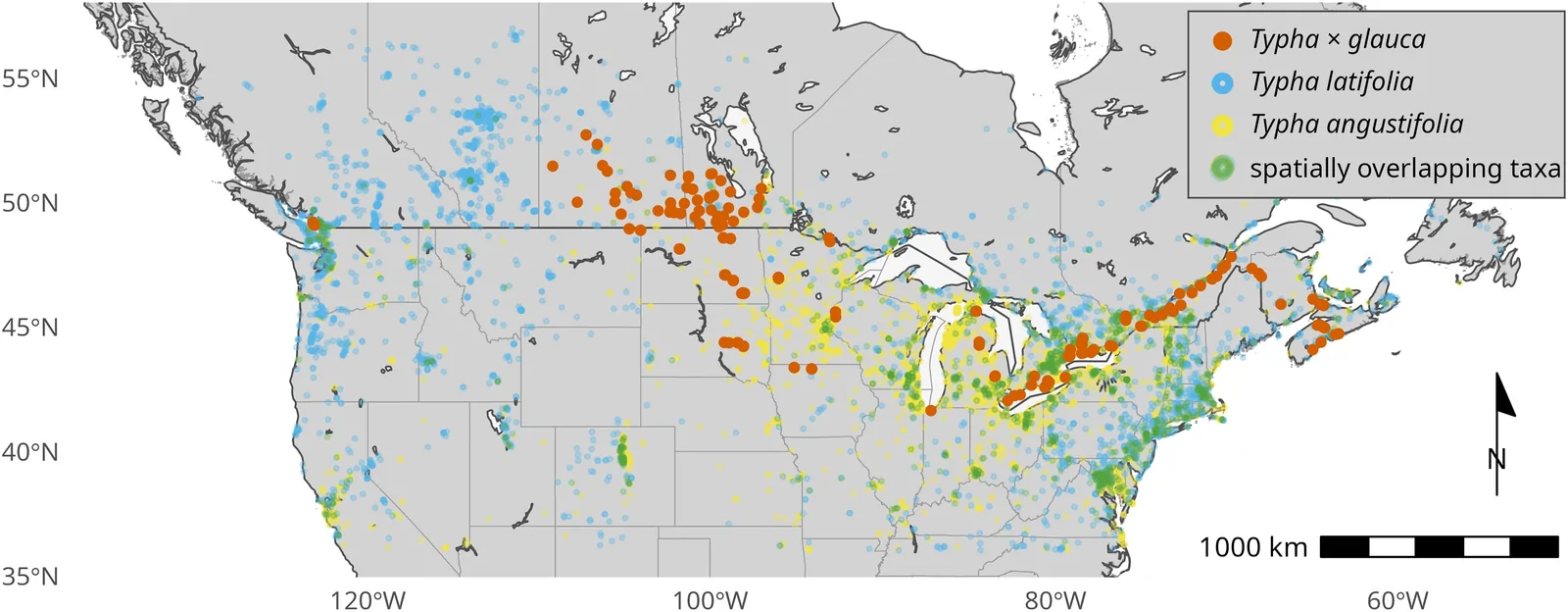
Map showing the known North American locations of *T. latifolia* (blue), *T. angustifolia* (yellow), and hybrids (F1 *T.* × *glauca* and non-F1 hybrids) The hybrid zone likely extends beyond this range, because data are limited to areas from which samples have been genotyped. Hybrid data are from Travis et al. (2010), Kirk et al. (2011), Freeland et al. (2013), Pieper et al. (2020), Tisshaw et al. (2020), Tangen et al. (2022), Joyee et al. (2024), and Buckholtz et al. (2025). Occurrence records of parental species (*T. angustifolia* and *T. latifolia*) were retrieved from the Global Biodiversity Information Facility Application Programming Interface (GBIF API) (GBIF.org 2026) using the rgbif R package (Chamberlain et al. 2026) and filtered by taxon name and the geographic extent included in the figure. Points were plotted using the ggblend R package (Kay 2025) to improve visibility of overlapping occurrences; overlap is shown by additive colour mixing (i.e. yellow + blue = green). See Tangen et al. (2022) for photos highlighting some key gross morphological differences between *T. latifolia, T. angustifolia*, and F1 *T. × glauca*.

Monitoring both the expansion and the ecological impacts of the *Typha* hybrid zone in North America is complicated by the fertility of F1 hybrids, which can interbreed with themselves (advanced-generation hybrids) and with their parent species (back-crossed hybrids) (Pieper et al. 2017); hereafter, we collectively refer to advanced-generation and back-crossed hybrids as non-F1 hybrids because differentiating hybrid classes in this complex hybrid zone is challenging (Freeland and Dorken 2026). Multiple studies that used genetic data to identify hybrids (F1 and non-F1) have shown that these are now the dominant *Typha* taxon in regions around the LGL, St. Lawrence Seaway, the upper Midwest USA, and the North American Prairie Pothole Region (Olson et al. 2009, Travis et al. 2010, Kirk et al. 2011, Freeland et al. 2013, Pieper et al. 2020, Tangen et al. 2022) (Fig. 2). Furthermore, the range of *T. × glauca* is continuing to expand (Stewart et al. 2023, Joyee et al. 2024). Genetic surveys have also shown that the abundance of non-F1s varies considerably among regions, comprising 50%–57% of ramets from the Midwestern USA and eastern North American Prairie Pothole Regions (Geddes et al. 2021, Tangen et al. 2022), but only 4%–17% of ramets from central and eastern Canada (Freeland et al. 2013).

While genetic data have provided valuable insights into the *Typha* hybrid zone (reviewed in Freeland and Dorken 2026), these methods are relatively expensive and inaccessible to some researchers and land managers. Multiple studies have therefore investigated whether morphological characters can reliably differentiate taxa, but concluded that there is substantial overlap of vegetative and other gross morphological characters between parent species and hybrids. Leaf width normally differentiates *T. latifolia* and *T. angustifolia*, but only in the absence of hybrids (Smith 1967, Grace and Harrison 1986, Tangen et al. 2022). *Typha* are monoecious, with the female and male inflorescences occurring on a single stem (spike), and studies that compared spike gap (distance between male and female inflorescences) among plants that had been identified to taxon from genetic data found that hybrids and parents overlapped in measurements of spike gap, spike width, and spike height (Tangen et al. 2022, Wasko et al. 2022). Differentiating *Typha* based on gross morphology is particularly challenging in regions where non-F1 hybrids are common because they collectively show greater morphological variability than both F1 hybrids and parent species (Tangen et al. 2022). Several measurements including mean leaf-apex angle, which measures the angle at the distal tip of the leaf (i.e. how pointy the leaf is) (Wasko et al. 2022), and combinations of vegetative characters (Ohsowski et al. 2024, Buckholtz et al. 2025) may be suitable for differentiating F1 hybrids from parent species, but these have so far been tested on only a few (<5) non-F1 hybrids, and it is therefore unclear how accurate these are in areas where non-F1 hybrids are common. For example, Geddes et al. (2021) found that a combination of leaf width and spike gap agreed with genetically-based taxonomic identifications for only 22% of hybrids in an area where non-F1 hybrids comprised ∼50% of the sampled plants. Furthermore, some gross morphological traits may have a degree of plasticity, e.g. one study found that leaf width was influenced by leaf length, nitrogen levels, and whether the shoot was flowering (Shapiro 2022).

Because no gross morphological characters have been shown to reliably differentiate F1 and non-F1 hybrids from parental species, we quantified the accuracy with which two microscopic reproductive traits—pollen and bracteoles—could identify species and hybrids. We did this by comparing genetic-based taxonomic identifications to bracteole- or pollen-based identifications using previously unpublished data. We then compared proportions of each taxon identified from samples of flowering shoots to those identified from randomized samples (i.e. sampled independently of flowering status) to investigate whether the proportion of flowering shoots is comparable among all taxa and thus whether using reproductive characters to identify plants might lead to biased estimates of the relative abundance of taxa. From these data, we asked the following: (i) What is the accuracy of microscopic reproductive-character based identification methods when tested against genotype-based taxon assignments? (ii) How concordant are taxonomic assignments based on pollen and bracteoles? (iii) To what extent might estimates of the relative frequencies of different cattail taxa (parental versus hybrid) be biased when using reproductive characters to identify plants?

## Materials and methods

Below we describe several previously unpublished datasets that allowed us to compare taxonomic assignments based on either pollen or bracteole morphology with those based on genetic data. These data are from (i) previously published studies that used morphological characters to provisionally identify plants in the field but did not report on the accuracy of these taxonomic identifications when compared with genotypes (Bhargav et al. 2022, Freeland et al. 2025); (ii) a study that identified plants based only on bracteoles and pollen (Melvin et al. 2024), a subset of which we subsequently genotyped; and (iii) comparisons of bracteole-based and genotype-based taxonomic identifications that have not previously been published. In each case, the genetic data comprise either multiple microsatellite loci with alleles that are specific to *T. angustifolia* or *T. latifolia* (described, along with methodology, in Kirk et al. 2011), or multiple SNP loci with alleles that are specific to either *T. angustifolia* or *T. latifolia* (described, along with methodology, in Chambers et al. 2024). Genotypes were based on a minimum of four independent loci, which is typically sufficient for differentiating F1 and non-F1 hybrids (Boecklen and Howard 1997). Plants were identified as *T. angustifolia* if they had only *T. angustifolia* alleles at all loci; *T. latifolia* if they had only *T. latifolia* alleles at all loci; F1 hybrids if they had one *T. angustifolia* allele and one *T. latifolia* allele at each locus; and non-F1 hybrids (advanced-generation or back-crossed) if they had only *T. latifolia* or *T. angustifolia* alleles at one or more loci plus a combination of *T. latifolia* and *T. angustifolia* alleles at one more loci (see Freeland and Dorken 2026, for a graphical explanation).

### Pollen

**Figure 3:**
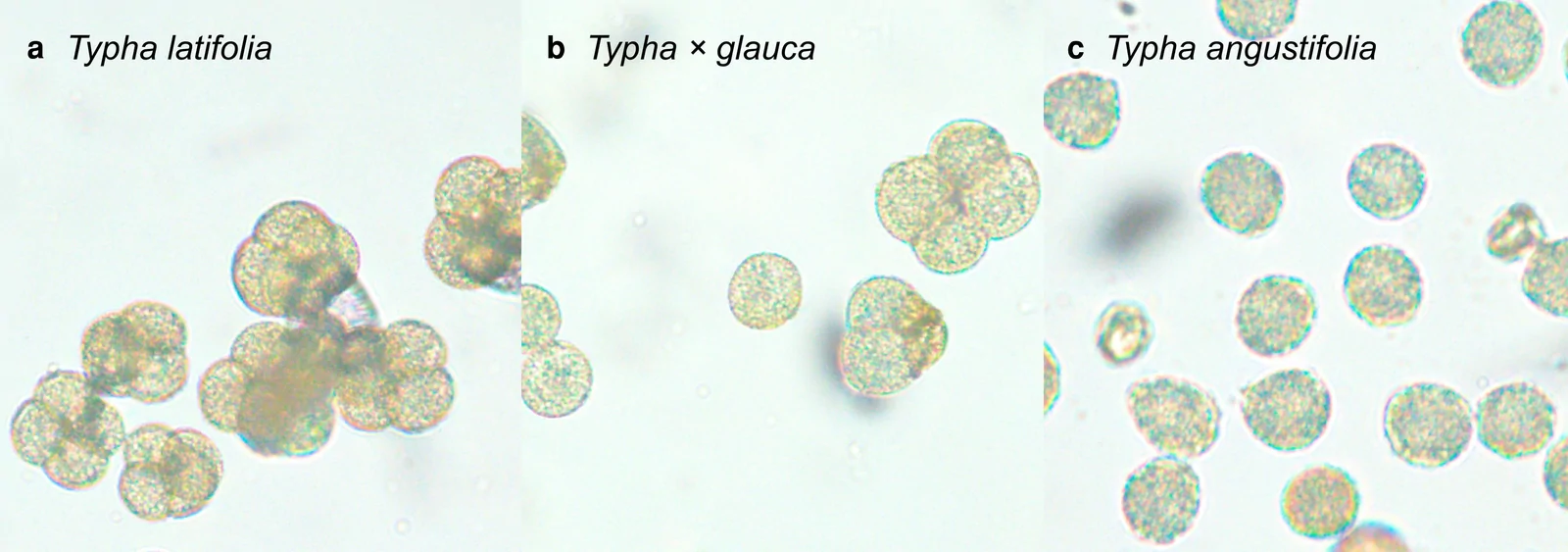
Magnified pollen from *T. latifolia, T. angustifolia*, and *T. × glauca* collected from Trent University campus, Peterborough, Ontario. a) *Typha latifolia* pollen sheds pollen in tetrads; b) *T. × glauca* pollen can shed a mixture of monads, dyads, triads, and tetrads; and c) *T. angustifolia* sheds pollen as monads. The pollen was viewed at 400× using a Nikon Eclipse e200 compound microscope (Nikon Canada, Mississauga, Ontario, Canada). Pollen was unstained and mounted on microscope slides in water.

Pollen grains can be useful for taxonomic assignments when the arrangement differs among taxa. *Typha latifolia* sheds pollen grains as tightly bound tetrads, whereas *T. angustifolia* has pollen grains arranged as monads, and those in F1 hybrids can be a mixture of monads, dyads, triads, and tetrads (Smith 1967, Dugle and Copps 1972, Finkelstein 2003) (Fig. 3). The arrangement of pollen grains in non-F1 hybrids has not been described, and thus we used pollen to assign plants to either *T. angustifolia*, *T. latifolia*, or hybrid (which could be F1 or non-F1). Each plant was also genetically identified as either *T. angustifolia*, *T. latifolia*, F1 hybrids, or non-F1 hybrids.

Here, we present previously unpublished results from three studies conducted within the hybrid zone that compared pollen-based and genotype-based taxonomic identifications. The first data are from a study by Bhargav et al. (2022), which tested the hypothesis of hybrid breakdown by using hand pollinations to generate hybrids in natural populations in and around Peterborough, Ontario, Canada. The plants used in these experiments were based on site surveys that targeted parental species and hybrids at multiple sites and provisionally identified plants to taxon based on pollen. These provisionally identified plants were later unequivocally identified to taxon based on four microsatellite loci (detailed methods are described in Bhargav et al. 2022), and any plants that were not genetically confirmed as the putative taxon were excluded from the study without reporting on the accuracy of the pollen-based identifications.

The second pollen data set is from a study that investigated the production of *T. angustifolia* versus hybrids in seeds collected from *T. angustifolia* (Freeland et al. 2025). This study of hybrid formation rates required fruit from open-pollinated maternal *T. angustifolia*, and initial identifications were based partly on pollen and subsequently verified using genetic data from five loci [one microsatellite locus and four single nucleotide polymorphisms (SNPs)]; this generated a dataset comparing pollen-based taxonomic assessments to genetic identification for *T. angustifolia* and hybrids (detailed methods are described in Freeland et al. 2025). As before, that study excluded plants that were not genetically confirmed as the putative taxon and did not report on the accuracy of the pollen-based identifications.

The third data set includes pollen-based taxonomic identifications from a study on *Typha* and muskrats (*Ondatra zibethicus*), in which researchers randomly sampled flowering shoots from multiple sites in southern Ontario in 2020 and 2021 (Melvin et al. 2024). The authors of that study identified taxa from a combination of pollen and bracteoles but did not include any genotyping. We genotyped at four SNP loci a subset (*n* = 54) of the plants sampled by Melvin et al. (2024) that were provisionally identified as hybrids from the pollen morphology, and this allowed us to assess the accuracy of pollen-based identifications.

### Bracteoles

**Figure 4:**
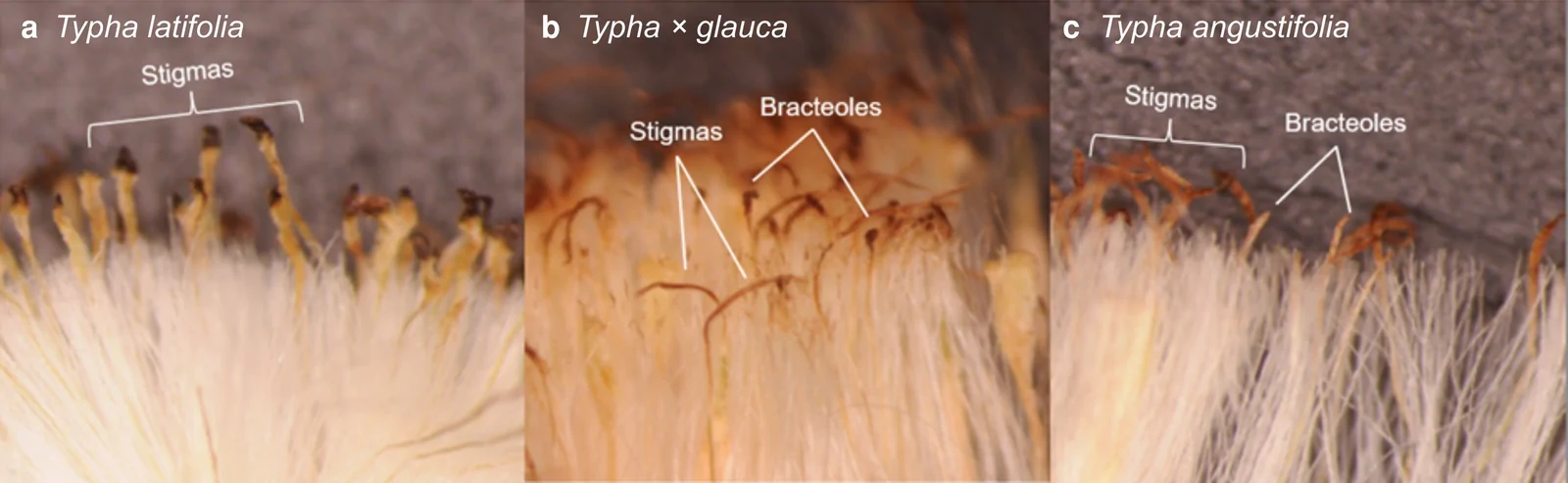
Magnified pistillate florets from *T. angustifolia* (left), *T. × glauca* (middle), and *T. latifolia* (right) showing the bracteoles and stigmas. *Typha angustifolia* exhibits darker bracteoles that are wider than the adjacent stigmas, while *T.* × *glauca* displays lighter-coloured bracteoles narrower than the adjacent stigmas. *Typha latifolia* lacks bracteoles. Bracteoles were viewed on a dissecting microscope (NIKON-SZ800) equipped with a Nikon D5000 DSLR camera (Tokyo, Japan) and a Fiber-Lite Mi-150 Illuminator (Dolan-Jenner, Boxborough, MA, USA).

Bracteoles are small bracts that in some cattail taxa occur on the female florets and are visible when viewed under a dissecting microscope. Smith (2000) included bracteoles in his *Typha* taxonomic key, stating that *T. latifolia* lacks bracteoles, *T. angustifolia* has dark bracteoles that are wider than adjacent stigmas, and *T.* × *glauca* has bracteoles that are lighter in colour and narrower than adjacent stigmas (Fig. 4). This key does not include comparisons of non-F1 hybrids, leaving a question about whether bracteoles are useful for identifying hybrid cattails in regions where non-F1 hybrids occur. Although we used plant material collected for earlier studies, those studies either did not incorporate bracteole characteristics into taxonomic identifications (plants collected for Bhargav et al. 2022, Joyee et al. 2024, Freeland et al. 2025) or did not have genotype-based taxonomic assignments to which the bracteole assignments could be compared (plants collected for Melvin et al. 2024).

We compared bracteole-based taxonomic identifications to genetic data for 244 plants sampled from Ontario, Saskatchewan, and Alberta, Canada (Table 1). Genetic data were the same as described above, i.e. a minimum of four loci (microsatellites and/or SNPs) with alleles specific to *T. angustifolia* or *T. latifolia* (Table 1). Morphological identifications followed the bracteole key of Smith (2000), using a small sample of florets (10–20) per inflorescence that were viewed at 20–40× on a dissecting microscope (NIKON-SZ800; Tokyo, Japan) (Fig. 4). Three parties were involved in this comparison: one to identify taxon based on bracteoles, one to identify taxon based on genotypes, and a third to independently compare the two sets of identifications. Each plant was identified as either *T. angustifolia*, *T. latifolia*, or hybrid (F1s and non-F1s combined) based on bracteoles, and as either *T. angustifolia*, *T. latifolia*, F1 hybrids, or non-F1 hybrids based on genetic data.

**Table 1:** Sources of samples used to compare bracteole-based and genotype-based taxonomic identifications of *T. latifolia*, *T. angustifolia*, F1 hybrids, and non-F1 hybrids.

Geographic region, year	Total number of plants and number of each taxon based on genotypes (citation refers to the study for which the samples were collected)	Genotypes
In and around Peterborough, Ontario, 2022	17: 0 *T. latifolia*, 13 *T. angustifolia*, 0 F1 hybrids, 4 non-F1 hybrids (Freeland et al. 2025)	Four SNP loci, one microsatellite locus (Freeland et al. 2025)
Western Prairie Pothole Region (Saskatchewan and Alberta, Canada), 2022	66:22 *T. latifolia*, 16 *T. angustifolia*, 24 F1 hybrids, 4 non-F1 hybrids (Joyee et al. 2024)	Four SNP loci, one microsatellite locus (Joyee et al. 2024)
In and around Peterborough, Ontario, 2019	24: 9 *T. latifolia*, 5 *T. angustifolia*, 6 F1 hybrids, 4 non-F1 hybrids (Bhargav et al. 2022)	Four microsatellite loci (Bhargav et al. 2022)
Southern central Ontario, 2021 and 2022	137: 4 *T. latifolia*, 18 *T. angustifolia*, 104 F1 hybrids, 11 non-F1 hybrids (Melvin et al. 2024)	Four SNP loci, one microsatellite locus (unpublished data)

The genotyped loci all have species-specific alleles (Kirk et al. 2011, Chambers et al. 2024).

### Concordance between bracteoles and pollen

Pollen-based and bracteole-based taxonomic identifications were compared for 274 plants collected from central Ontario in 2021 and 2022 in an area that includes *T. latifolia*, *T. angustifolia*, and hybrids (Melvin et al. 2024). This comparison did not differentiate F1 from non-F1 hybrids. The goal here was to test the agreement between the two characters, because a high degree of concordance should increase confidence in using these traits. Additionally, genetic data were available for 54 of these plants, which allowed us to compare taxonomic identifications from pollen, bracteoles, and genotypes for this subset.

### Flowering shoots across taxa

The utility of inflorescence characters for taxonomic assignment may also depend on the proportion of shoots that are flowering, and we investigated whether identifying plants from flowering traits may bias *Typha* taxonomic conclusions. We compared proportions of different taxa sampled from transects in southern Ontario that have been reported in two previously published studies. In one study, plants were sampled regardless of whether they were flowering (*n* = 547 plants across six sites; Pieper et al. 2020), whereas in the second study, plants were sampled along transects only if they were flowering (*n* = 152 across three sites). Plants in the first study were assigned to taxon on the basis of four microsatellite loci with species-specific alleles (Pieper et al. 2020, following the methods of Kirk et al. 2011), and plants in the second study were assigned to taxon based on four loci (microsatellite and/or SNPs) with species-specific alleles (unpublished data, following the methods of Chambers et al. 2024).

## Results

### Pollen

In the study by Bhargav et al. (2022), genotypes confirmed the pollen-based taxonomic identification for 215/216 hybrids, 51/53 *T. angustifolia*, and 38/42 *T. latifolia*, leading to an overall accuracy of 304/311 = 97.7% for pollen-based identifications. The seven plants that were incorrectly identified from pollen were all genetically identified as either non-F1 hybrids (*n* = 7) or *T. angustifolia* (*n* = 1). In the study by Freeland et al. (2025), genetic identification of 61 plants identified from pollen as *T. angustifolia* showed an accuracy of 53/61 plants (86.9%). Again, all incorrectly identified plants were identified as non-F1 hybrids using genetic methods. Pollen-based taxonomic assignments from Melvin et al. (2024) agreed with genotype-based taxonomic assignments for 52/54 (∼96%) of the genotyped plants. For this study, incorrectly identified plants comprised one non-F1 hybrid and one *T. angustifolia*. Combining the results of these three studies gives an overall concordance of 409/426 (96%) between genetic and pollen-based taxonomic identification for *T. latifolia*, *T. angustifolia* and hybrids, with the lowest match for non-F1 hybrids (Table 2).

**Table 2:** Summary of genetic-based taxonomic identification compared with pollen-based taxonomic identification of *T. latifolia*, *T. angustifolia*, F1 *T.* × *glauca*, and non-F1 hybrids.

Genetic-based taxonomic identification	Percent scored as *T. latifolia* based on pollen identification	Percent scored as *T. angustifolia* based on pollen identification	Percent scored as hybrids based on pollen identification
*Typha latifolia*	**100%** (**40)**	0%	0%
*Typha angustifolia*	0%	**99% (113)**	1% (1)
*F1 T. × glauca*	0%	0%	**100% (227)**
Non-F1 hybrids	9% (4)	27% (12)	**64% (29)**

Bold text identifies the proportion of plants with taxonomic agreement based on pollen and genotypes.

### Bracteoles

The genetic analysis of the plants that were provisionally identified to taxon using bracteole characters revealed that 35 were *T. latifolia*, 52 were *T. angustifolia*, 134 were F1 *T.* × *glauca*, and 23 were non-F1 hybrids. Because we did not differentiate F1 from non-F1 hybrids based on bracteoles, plants that were genetically identified as non-F1 hybrids were considered accurately identified from bracteoles if they were assigned as hybrids. Overall, genetic-based and bracteole-based taxonomic identification agreed for 232/244 (95%) plants, although this varied considerably across taxonomic groups. There was 100% agreement between bracteole- and genetic-based taxonomic assignment of the 35 *T. latifolia* plants and of the 134 F1 *T.* × *glauca* plants. Two of the 52 plants identified as *T. angustifolia* from genetic data were misidentified as hybrids (accuracy of ∼96%) using bracteole morphology. Non-F1 hybrids had the smallest percentage of matches (13/23 = 56.5%) between bracteole- and genetic-based identification: nine of the genetically identified non-F1 hybrids were identified as *T. angustifolia* based on bracteoles, and one as *T. latifolia* (Table 3).

**Table 3:** Summary of genetic-based taxonomic identification compared with bracteole-based taxonomic identification of *T. latifolia*, *T. angustifolia*, F1 *T.* × *glauca*, and non-F1 hybrids.

Genetic-based taxonomic identification	Percent scored as *T. latifolia* based on bracteole identification	Percent scored as *T. angustifolia* based on bracteole identification	Percent scored as hybrids based on bracteole identification
*Typha latifolia*	**100%** (**35)**	0%	0%
*Typha angustifolia*	0%	**96% (50)**	4% (2)
*F1 T. × glauca*	0%	0%	**100% (134)**
Non-F1 hybrids	4% (1)	39% (9)	**57% (13)**

Bold text identifies the proportion of plants with taxonomic agreement based on bracteoles and genotypes.

### Concordance between bracteoles and pollen

Pollen-based and bracteole-based taxonomic identifications agreed for 262/274 (95.6%) plants: 12 *T. latifolia*, 17 *T. angustifolia*, and 233 hybrids. Fifty-three (98%) of the taxonomic assignments in the 54 plants for which we had pollen, bracteole, and genotype taxonomic identifications were in agreement; the remaining plant was identified as *T. angustifolia* based on both pollen and bracteoles but as a non-F1 hybrid based on genetic data.

### Flowering shoots across taxa

The proportion of taxa varied substantially between studies: when all shoots were sampled regardless of flowering (Pieper et al. 2020), genetic data showed that they comprised 101 (18%) *T. latifolia*, 80 (15%) *T. angustifolia*, 315 (58%) F1 *T.* × *glauca*, and 51 (9%) non-F1 hybrid shoots. However, when only flowering shoots were sampled (Melvin et al. 2024), the genotypes revealed 7 *T. latifolia* (5%), 24 *T. angustifolia* (16%), 110 F1 *T.* × *glauca* (72%), and 11 non-F1 hybrids (7%). A chi-square test of independence revealed a significant difference in the frequencies of the four taxa between the randomly collected versus flowering-shoot samples (*χ*^2^ = 19.75, *df* = 3, *n* = 699, *P* = <0.0001).

## Discussion

The *Typha* hybrid zone in North America comprises *T. latifolia*, *T. angustifolia*, F1 *T. × glauca* hybrids, and non-F1 (advanced-generation and back-crossed) hybrids. The full extent of this hybrid zone is unknown but likely covers >2 million km^2^ (Olson et al. 2009, Travis et al. 2010, Kirk et al. 2011, Freeland et al. 2013, Geddes et al. 2021, Tangen et al. 2022, Joyee et al. 2024). Challenges with differentiating the hybrid from parental species based on morphological characters, particularly when non-F1 hybrids co-occur with parent species, have in some cases hampered our understanding of range expansions and areas across which hybrids are invasive. This may be particularly problematic in areas of high non-F1 hybrid abundance.

We summarized—and in one case supplemented—unpublished data that were originally collected for three previously published studies to show high taxonomic concordance between genotypes and pollen (average of ∼96%). The highest error rate involved non-F1 hybrids, although a sufficient sample size should allow researchers to determine whether hybrids are present at a site even if there is a high proportion of non-F1 hybrids. However, there are two limitations to this trait. The first is the need for well-timed visits, because in the Great Lakes region pollen-shedding typically lasts for ∼2–6 days at the individual level, and within sites (across taxa) can last for 16–68 days (Ball and Freeland 2013, Selbo and Snow 2004). The second limitation is that many *Typha* do not flower each year; e.g. only 49% (22 of 45) and 27% (20 of 75) of the ramets sampled by Wasko et al. (2022) and Ohsowski et al. (2024), respectively, had an inflorescence.

We have also presented novel data showing that concordance between genotype-based and bracteole-based taxonomic identifications is high (average of 95%), with the highest error rate again involving non-F1 hybrids. Our data show that an absence of bracteoles is a reliable trait for identifying *T. latifolia* and that bracteoles can be used to differentiate *T. angustifolia* and F1 hybrids with a high degree of accuracy. Although bracteoles are much less reliable for differentiating *T. angustifolia* and non-F1 hybrids, a sufficient sample size should allow researchers to determine whether hybrids are present at a site even when non-F1 hybrids predominate. Bracteoles have a much longer sampling window compared to pollen: while some fruit may be shed at the end of the growing season, other plants retain fruit into the following spring. However, there is still the limitation that not all *Typha* ramets produce flowers. Furthermore, our data suggest that the proportion of flowering ramets can differ among taxa, potentially introducing another bias into taxonomic identification based on inflorescence characteristics. More specifically, our comparison between studies shows that the abundance of *T. latifolia* may be underestimated if plants are identified from inflorescence traits.

Overall, our data show that microscopic reproductive characters are highly effective at discriminating parental and hybrid cattails; however, our results also reiterate that the occurrence of non-F1 hybrids complicates the identification of cattail taxa in the North American hybrid zone. Other nongenetic methods that have been proposed for identifying cattail taxa have so far been tested on very small numbers of non-F1 hybrids (Wasko et al. 2022, Ohsowski et al. 2024). As a result, in regions where non-F1 hybrids are common (e.g. central Canada; Freeland et al. 2013) or dominant (e.g. Midwestern USA and eastern North American Prairie Pothole Regions (Geddes et al. 2021, Tangen et al. 2022), it is unclear whether cattails are likely to be identified accurately using either the reproductive characters studied here or methods based on vegetative morphology (e.g. Ohsowski et al. 2024). It would be valuable to compare these methodologies in the future—possibly combining them into a multivariate analysis—and validate them with genotype-based identifications. These types of analyses would clarify the extent to which nongenetic approaches are useful for identifying cattails when discrimination among taxa—particularly between non-F1 hybrids and *T. angustifolia*—is important.

More broadly, our results identify challenges that may extend to many different hybrid zones. Gross morphological characters can be useful for identifying F1 hybrids when traits are intermediate to those of their parent species (e.g. Fukami et al. 2019, Thomasset et al. 2011), although cryptic hybrids that closely resemble one or both parent species are more common in some taxa, and these often require some preliminary investigations using genetic data (e.g. Mitchell and Holsinger 2018, Yi et al. 2023). Data collected so far from the North American *Typha* hybrid zone suggest that although parent species can be differentiated from one another and from F1 hybrids using a suite of different characters, differentiating non-F1 hybrids from *T. angustifolia* is challenging when using either gross morphological or microscopic characters. Overall, non-F1 hybrids are genetically more similar to *T. angustifolia* than to *T. latifolia*, possibly because hybrid backcrosses to *T. angustifolia* are relatively common (Freeland and Dorken 2026), and this could help to explain some of the challenges differentiating non-F1 hybrids from *T. angustifolia*. Findings such as this reiterate the need in newly described hybrid zones to ensure that all potential hybrid classes are considered when determining which characteristics are useful for identifying taxa: while some hybrid zones are limited to parental species and F1 taxa, more complex hybrid zones may require extensive research on hybrid fertility and the potential for different hybrid classes (F1s, advanced-generation hybrids, and back-crossed hybrids). In regions of the North American *Typha* hybrid zone where non-F1 hybrids are relatively uncommon, managers may find pollen and bracteoles more informative than gross morphological characters for differentiating taxa; however, in regions with high proportions of non-F1 hybrids, larger sample sizes of flowering plants may be needed in order to ensure that at least some non-F1 hybrids are correctly identified.

## Supplementary Material

plag035_Supplementary_Data

## Data Availability

Data are summarized in Tables 1, 2, and 3 and in the Supplementary material.
